# Supplementary material for: The association between limiting longstanding illness and serious psychological distress in adolescents: A secondary analysis of the UK Millennium Cohort Study
Source: PLoS One. 2025 Sep 8;20(9):e0306677. doi: 10.1371/journal.pone.0306677 (PMC12416657; doi:10.1371/journal.pone.0306677)
Supplement: S1 File — Supplementary appendices containing (S1) Weighted summary statistics of covariates by LLSI status in complete case dataset; (S2) Unweighted complete-case multiple logistic regression for dichotomised Kessler-6 score; (S3) Weighted multiple logistic regression of dichotomised Kessler-6 score using Multiple Imputation by Chained Equations; (S4) Unweighted complete-case multiple logistic regression of currently receiving treatment for depression or serious anxiety. (DOCX) [file pone.0306677.s001.docx]

The association between limiting longstanding illness and serious psychological distress in adolescents

**Appendix S1**

Weighted summary statistics of covariates by LLSI status in complete case dataset

**Appendix S2**

Unweighted complete-case multiple logistic regression for dichotomised Kessler-6 score

**Appendix S3**

Weighted multiple logistic regression of dichotomised Kessler-6 score using Multiple Imputation by Chained Equations

**Appendix S4**

Unweighted complete-case multiple logistic regression of currently receiving treatment for depression or serious anxiety

Appendix S1:

Weighted summary statistics of covariates by LLSI status in complete case dataset

| Variable | No LLSI (%) | LLSI (%) | Total  (%) |
| --- | --- | --- | --- |
| Categorical |  |  |  |
| Kessler-6 dichotomised score (primary outcome) |  |  |  |
| No serious psychological distress | 4710 (85.4) | 916 (79.2) | 5626 (84.3) |
| Serious psychological distress | 807 (14.6) | 241 (20.8) | 1048 (15.7) |
| Currently receiving treatment for anxiety or depression (secondary outcome) |  |  |  |
| No | 5322 (96.5) | 1027 (88.8) | 6349 (95.2) |
| Yes | 194 (3.5) | 129 (11.2) | 323 (4.8) |
| Sex |  |  |  |
| Female | 2833 (51.4) | 522 (45.1) | 3355 (50.3) |
| Male | 2683 (48.6) | 635 (54.9) | 3317 (49.7) |
| Ethnic group |  |  |  |
| White | 5013 (90.9) | 1052 (91.0) | 6065 (90.9) |
| Indian | 80 (1.5) | 19 (1.6) | 99 (1.5) |
| Pakistani and Bangladeshi | 115 (2.1) | 19 (1.7) | 135 (2.0) |
| Black or Black British | 109 (2.0) | 18 (1.6) | 128 (1.9) |
| Mixed or Other Ethnic group | 199 (3.6) | 48 (4.2) | 247 (3.7) |
| Family structure |  |  |  |
| Two parents/carers | 4876 (88.4) | 953 (82.4) | 5828 (87.3) |
| Single parent/carer | 641 (11.6) | 204 (17.6) | 844 (12.7) |
| Parental education |  |  |  |
| None or overseas qualification | 510 (9.2) | 136 (11.8) | 646 (9.7) |
| NVQ level 1 | 419 (7.6) | 112 (9.7) | 532 (8.0) |
| NVQ level 2 | 1620 (29.4) | 341 (29.5) | 1961 (29.4) |
| NVQ level 3 | 818 (14.8) | 164 (14.2) | 982 (14.7) |
| NVQ level 4 | 1918 (34.8) | 354 (30.7) | 2273 (34.1) |
| NVQ level 5 | 231 (4.2) | 48 (4.2) | 279 (4.2) |
| Parental limiting longstanding illness |  |  |  |
| No | 4442 (80.5) | 817 (70.6) | 5258 (78.8) |
| Yes | 1075 (19.5) | 340 (29.4) | 1415 (21.2) |
| OECD equivalised income |  |  |  |
| Above 60% median | 4228 (76.7) | 783 (67.7) | 5011 (75.1) |
| Below 60% median | 1288 (23.4) | 374 (32.3) | 1662 (24.9) |
| Continuous | **No LLSI**  **mean (SD)** | **LLSI**  **mean (SD)** | **Total**  **mean (SD)** |
| Parental Kessler-6 score | 2.90 (0.63) | 4.04 (0.18) | 3.10 (0.06) |

Appendix S2

Unweighted complete-case multiple logistic regression of dichotomised Kessler-6 score

| **Covariate** |  | | |  |
| --- | --- | --- | --- | --- |
|  | **Unweighted adjusted OR** | **95% CI** | **P-value** | |
| **Longstanding illness** |  |  |  | |
| No longstanding illness | 1.00 |  | (ref) | |
| Longstanding illness | 1.64 | 1.41-1.91 | <0.001 | |
| **Sex** |  |  |  | |
| Female | 1.00 |  |  | |
| Male | 0.39 | 0.34-0.44 | <0.001 | |
| **Ethnic group** |  |  |  | |
| White | 1.0 |  | (ref) | |
| Indian | 0.62 | 0.37-1.04 | 0.070 | |
| Pakistani/ Bangladeshi | 0.62 | 0.44-0.89 | 0.009 | |
| Black/ Black British | 0.69 | 0.44-1.10 | 0.120 | |
| Mixed/ Other | 1.07 | 0.77-1.47 | 0.697 | |
| **Family structure** |  |  |  | |
| Dual parents/carers | 1.00 |  | (ref) | |
| Single parent/carer | 1.00 |  |  | |
| **Parental education** |  |  |  | |
| None or overseas only | 1.00 |  | (ref) | |
| NVQ level 1 | 1.01 | 0.76-1.35 | 0.924 | |
| NVQ level 2 | 0.99 | 0.80-1.24 | 0.962 | |
| NVQ level 3 | 1.00 | 0.78-1.29 | 0.982 | |
| NVQ level 4 | 0.83 | 0.66-1.05 | 0.128 | |
| NVQ level 5 | 0.87 | 0.60-1.25 | 0.452 | |
| **OECD equivalised income** |  |  |  | |
| Above 60% median | 1.00 |  | (ref) | |
| Below 60% median | 1.17 | 0.99-1.38 | 0.066 | |
| **Parental limiting longstanding illness** |  |  |  | |
| No | 1.00 | 0.068 | (ref) | |
| Yes | 1.15 | 0.99-1.33 | 0.068 | |
| **Parental Kessler-6 score** | 1.04 | 1.02-1.06 | <0.001 | |
| **Constant** | 0.22 | 0.18-0.28 | <0.001 | |

*Cutoff score of* ≥*13 on the Kessler-6 used to indicate serious psychological distress*

Appendix S3:

Weighted multiple logistic regression of dichotomised Kessler-6 score using Multiple Imputation by Chained Equations

| **Covariate** |  | |  |  |
| --- | --- | --- | --- | --- |
|  | **Unweighted adjusted OR** | **95% CI** | | **P-value** |
| **Longstanding illness** |  |  | |  |
| No longstanding illness | 1.00 |  | | (ref) |
| Longstanding illness | 1.57 | 1.36-1.84 | | <0.001 |
| **Sex** |  |  | |  |
| Female | 1.00 |  | |  |
| Male | 0.38 | 0.33-0.43 | | <0.001 |
| **Ethnic group** |  |  | |  |
| White | 1.00 |  | | (ref) |
| Indian | 0.64 | 0.41-0.99 | | 0.048 |
| Pakistani/ Bangladeshi | 0.57 | 0.41-0.78 | | 0.001 |
| Black/ Black British | 0.55 | 0.37-0.83 | | 0.006 |
| Mixed/ Other | 0.98 | 0.68-1.40 | | 0.819 |
| **Family structure** |  |  | |  |
| Dual parents/carers | 1.00 |  | | (ref) |
| Single parent/carer | 1.07 | 0.89-1.28 | | 0.481 |
| **Parental education** |  |  | |  |
| None or overseas only | 1.00 |  | | (ref) |
| NVQ level 1 | 0.97 | 0.70-1.35 | | 0.851 |
| NVQ level 2 | 0.95 | 0.78-1.16 | | 0.621 |
| NVQ level 3 | 0.99 | 0.76-1.28 | | 0.912 |
| NVQ level 4 | 0.83 | 0.64-1.07 | | 0.146 |
| NVQ level 5 | 0.80 | 0.58-1.11 | | 0.187 |
| **OECD equivalised income** |  |  | |  |
| Above 60% median | 1.00 |  | | (ref) |
| Below 60% median | 1.18 | 1.00-1.40 | | 0.047 |
| **Parental limiting longstanding illness** |  |  | |  |
| No | 1.00 |  | | (ref) |
| Yes | 1.08 | 0.92-1.26 | | 0.351 |
| **Parental Kessler-6 score** | 1.04 | 1.02-1.06 | | <0.001 |
| **Constant** | 0.24 | 0.20-0.31 | | <0.001 |

Appendix S4:

Unweighted complete-case logistic regression of currently receiving treatment for depression or serious anxiety

| **Covariate** | **Adjusted** | | | |
| --- | --- | --- | --- | --- |
|  | **OR** | **P-value** | **95% CI** | |
| **Longstanding illness** |  |  |  | |
| No longstanding illness | 1.00 | (ref) |  | |
| Longstanding illness | 2.88 | <0.001 | 2.24-3.71 | |
| **Sex** |  |  |  |  |
| Female | 1.00 | (ref) |  |  |
| Male | 0.31 | <0.001 | 0.24-0.41 | |
| **Ethnic group** |  |  |  |  |
| White | 1.00 | (ref) |  | |
| Indian | 0.47 | 0.202 | 0.15-1.50 | |
| Pakistani/ Bangladeshi | 0.30 | 0.021 | 0.11-0.84 | |
| Black/ Black British | 0.49 | 0.229 | 0.15-1.57 | |
| Mixed/ Other | 0.26 | 0.024 | 0.08-0.84 | |
| **Family structure** |  |  |  |  |
| Dual parents/carers | 1.00 | (ref) |  | |
| Single parent/carer | 0.85 | 0.447 | 0.55-1.30 | |
| **Parental education** |  |  |  |  |
| None or overseas only | 1.00 | (ref) |  | |
| NVQ level 1 | 1.46 | 0.153 | 0.87-2.47 | |
| NVQ level 2 | 0.89 | 0.619 | 0.58-1.39 | |
| NVQ level 3 | 0.71 | 0.185 | 0.42-1.12 | |
| NVQ level 4 | 0.71 | 0.140 | 0.47-1.12 | |
| NVQ level 5 | 1.19 | 0.587 | 0.64-2.22 | |
| **OECD equivalised income** |  |  |  |  |
| Above 60% median | 1.00 | (ref) |  | |
| Below 60% median | 0.99 | 0.939 | 0.71-1.37 | |
| **Parental limiting longstanding illness** |  |  |  |  |
| No | 1.00 | (ref) |  | |
| Yes | 1.46 | 0.005 | 1.12-1.89 | |
| **Parental Kessler-6 score** | 1.06 | <0.001 | 1.03-1.09 | |
| **Constant** | 0.05 | <0.001 | 0.03-0.08 | |
