# Supplementary material for: The association between limiting longstanding illness and serious psychological distress in adolescents: A secondary analysis of the UK Millennium Cohort Study
Source: PLoS One. 2025 Sep 8;20(9):e0306677. doi: 10.1371/journal.pone.0306677 (PMC12416657; doi:10.1371/journal.pone.0306677)
Supplement: Attachment — Submitted filename: Review PlosOne.docx [file pone.0306677.s002.docx]

**PLOS ONE**

**The association between limiting longstanding illness and serious psychological**

**distress in adolescents: A secondary analysis of the UK Millennium Cohort Study**

**Reviewer comments:**

**Summary of the research**

This is an interesting and meaningful study of the relationship between having a serious, incurable long-standing medical (physiological) illness (referred to as a limiting longstanding illness; **LLSI**), and the concurrent development of a serious mental disorder.

The authors later adapt the definition of an LLSI such that the person does not need to have a long-standing physical/medical condition. Rather, a 17 year-old person with a diagnosed, medicated mental disorder is considered to have an LLSI. This aspect of the study is less clear and less well-explained, as anxiety and depression are not considered incurable conditions. There are no clear parameters outlined to explain why this condition is also an LLSI. Information is lacking regarding the length of the condition. It appears that anyone with anxiety and taking meds is automatically an LLSI. This is an element of the study that needs better clarification.

The authors use available data from a large, UK Millennium Cohort database.

**The research question is:** “This study therefore explored the association between having any LLSI before aged 17 years with serious psychological distress at age 17 years in a large representative UK birth cohort- the Millennium Cohort Study. This study also investigated the relationship between LLSI and receiving treatment for depression or serious anxiety.”

Major Issues:

1. The study is interesting, and the writing is good. The authors could try to make the paper more readable to the audience, so that readers clearly understand what the authors wish to convey. It is a worthwhile effort, as the study is important. The study should be very clearly understood. Sometimes the explanations are not very clear. Long sentences are not very helpful.
2. **Lines 66-69**. Here the authors are looking at a LLSI requiring medical appointments, less opportunity to engage in activities, and how these can add to one’s burden via the development of a mental disorder. “It is important to understand any association between physical and mental illnesses to inform prevention strategies...”

**Lines 77-80**. The study is described as looking at the association of young people with some physical illness (e.g., asthma, diabetes), along with a mental disorder. But later (see below, **lines 132-136)** **they suggest that if the child has anxiety or depression, and is being treated for it, then this is considered an LLSI.** This comes across as inconsistent with the earlier definition of an LLSI, and the intention to evaluate the presence of a medical condition with a mental disorder. This needs to be made clearer. There should be a clear justification and rationale for presuming your conclusion.

3. Also, later **(lines 147 and 148)** the authors state that those with only a mental health condition and nothing else were **not** considered to have an LLSI. Yet they then include mental disorders as a form of LLSI. So again, this becomes somewhat confusing for the readers. It would be helpful to make this very clear, and your claims should be consistent and justified.

The provided definition of an LLSI by the UK Department of Health is **[Lines 59-61]**: “any illness or condition which cannot be cured, but can instead be treated with pharmacological or other treatments.” How do anxiety or depressive disorders fit into this? Do you see these disorders as permanent, as unable to be cured? If so, this does not seem to be logical. Someone could be on medicine for only 2 weeks or 1 month for anxiety, but this does not suggest and LLSI. And a multitude of people have been able to overcome both of these disorders.

So it is difficult to see how the authors are justifying this aspect of the study as both of these mental disorders can be “cured.” So please explain this clearly so that the reader can understand your rationale for referring to these disorders as incurable and as being considered an LLSI after simply being given a diagnosis and a prescription.

Minor Issues:

1. The authors begin some sentences with numbers or numerical percentages. A sentence should always be started with written-out **words**, and **not with numbers** unless you wish to write-out the numbers. This is a clear requirement in the BPA (British Psyc Assoc: <https://cms.bps.org.uk/sites/default/files/2022-07/BPS%20Style%20Guide.pdf>) AMA, the APA. You can either write out the number or begin the sentence another way. For example:

**Example:**

**Line 216**: You write: “21.9% (302) of those with an LLSI scored above the threshold for serious psychological distress on the Kessler-6 compared with 14.5% (938) of those without an LLSI.” You have two options and here is a demonstration:

a) Twenty-one point nine percent of those with an LLSI scored….. [but this is a bit awkward]
b) Compared to the 14.5% (*N*=938) with no LLSI, those with an LLSI (21.9%; *N*=302) scored above the threshold for serious psychological distress on the Kessler-6.

**Lines 121 – 126** – the authors suggest that a cut off score of **12** on the K6 considers children with that score, from **12 or lower**, to **not have psychological distress**. Those with a score of 13 and higher are considered having “serious distress.” However,

1. The authors then suggest that those with scores of **12 – 24 have “serious psychological distress.”** But the score of “**12”** was just noted prior to this as representing no distress. So it cannot serve as an indicator of “no distress,” and simultaneously as an indicator of “serious psychological distress.” Please correct this.

**Lines 208 to 211**. The authors write: “Those with a reported LLSI also saw slightly higher prevalence of LLSI in proportions of those from single parent families, those below the 60% median equivalised household income threshold, and those from the White ethnic group, although the study population is also overwhelmingly white at 88.1%.”

It’s a long sentence. Do you mean to say:

“The prevalence of LLSIs was slightly higher in single-parent families and those with lower income (below 60% median). LLSIs were also higher among white ethnic persons, yet this is most likely due to most of participants being white (88.2%).”

**It is helpful to write sentences that are clear, crisp, concise.**

**Line 201**. Not all readers will understand your use of “weighted” and “unweighted” data. Also, when you provide the statistical scores related to the OR’s, these should be interpreted for the reader so that they understand what this means.

For example, you write: “The crude OR for children and adolescents who reported currently receiving treatment for depression or serious anxiety by age 17 years in those with an LLSI compared to those without was 3.18…” Explain to the reader what this actually means; apply it to one’s life (e.g., this means that those youth w/ XXX condition were more than three times more likely to……” ; otherwise, some readers will not grasp the meaning and importance of these statistics.

Before using OR (the abbreviation), state it out like this: **Odds Ratio (OR).** If you have done this already and I missed it, then ok. Otherwise, everything in a scientific paper should be written out clearly for the reader. This is to increase readability, understanding, and appreciation for the study.

**Lines 254, 255.** It is somewhat risky to imply that study should be considered as presenting **causal** results, especially given the imputation of some data, and that it is a “universal” data-base. While such data-bases are certainly helpful and useful, given the unknowns (who collected the data, level of experience and training in data collection, etc), we cannot presume more than what they were intended for. However, the findings are important, and future research could include a current study with children and adolescents, face-to-face.

**Lines 266-269.** Nice to add this here.

**Lines 333-335.** Important that you included this. There are many controversial views about imputing data, and some (perhaps most) use the easiest method which can certainly alter the results of a study. Higher level methods are more reliable.

Overall, there are very good things about this paper. Also, the references are strong. The paper can be improved by addressing the issues outlined above.

Wishing the authors all the best.
