# Supplementary material for: The association between limiting longstanding illness and serious psychological distress in adolescents: A secondary analysis of the UK Millennium Cohort Study
Source: PLoS One. 2025 Sep 8;20(9):e0306677. doi: 10.1371/journal.pone.0306677 (PMC12416657; doi:10.1371/journal.pone.0306677)
Supplement: Attachment — Submitted filename: Response to reviewers duplicate.docx [file pone.0306677.s004.docx]

Dear editorial team,

Thank you for the opportunity to revise our manuscript, *The association between limiting longstanding illness and serious psychological distress in adolescents: A secondary analysis of the UK Millennium Cohort Study*, and for the helpful comments from your reviewers. We have revised the manuscript in the light of these and respond point by point to the reviewers’ comments below.

Yours faithfully,

Stuart Jarvis

| **Reviewer 1 comments** | **Response to reviewer 1** |
| --- | --- |
| I appreciate the opportunity to review your manuscript, which presents an important examination of LLSI and psychological distress at age 17. I found the paper compelling, but I have a few suggestions that I believe would strengthen the clarity and flow of the manuscript. | We thank the reviewer for their comments and constructive suggestions for improvement |
| In Line 29, the phrase “self-reported currently receiving treatment for depression or serious anxiety” felt a bit clunky and disrupted the flow of the text. I understand the intent behind capturing the self-reported aspect of treatment, but I wonder if there might be a more concise way to phrase this. | We agree that this was hard to read and have revised it to “self-reported current treatment for depression or serious anxiety”, |
| I also noticed areas where the flow could be improved by adjusting the placement of citations or statistics. For example, in Line 94, shifting the reported odds to follow "increased odds" might make the sentence easier to read. | We have revised to “…those with a chronic illness had increased likelihood (OR 1.6, 95%CI 1.14-2.25) of having mental illness…”, which we believe flows better. |
| Similarly, I observed that citations are often placed at the beginning of sentences — in some cases, it might be more helpful to cite studies closer to the outcome statement, especially when referencing multiple sources. That said, I recognize that these are stylistic preferences, so please feel free to disregard if not relevant to your chosen writing style. | We have made some edits to move references to more appropriate places within sentences. |
| One area where I think the manuscript could benefit from further development is in the introduction. While you make a strong case for examining LLSI and distress, the importance of exploring whether treatment was being received is less clearly articulated. Expanding on this point would help reinforce the rationale for including treatment status as a key variable. | Thank you – we think this is partly influenced by our (now corrected) error in wording on the outcomes (please see next point, below) but have also added an explicit rationale for including this outcome to the end of the introduction: “to explore not only any links between LLSI and psychological distress, but also associations with related healthcare use and to explore any differences between presence of psychological distress and access to related treatment.” |
| I also had some difficulty understanding the definition and classification of LLSI, and there seemed to be some discrepancies from the introduction to the methods section. In Line 134, the statement that participants answering "yes" to both questions were considered to have an LLSI seemed to suggest that receiving treatment might be a criterion for LLSI. This was a bit confusing, as I had understood LLSI to refer to chronic illness limiting activities, with treatment status as a separate variable so that associations could be examined. If treatment is not a criterion for LLSI, clarifying this distinction in the methods section would be helpful to avoid misinterpretation. | Thank you. There was a mistake in wording here, mixing up the exposure (LSLI) with the second outcome of treatment for depression or serious anxiety. These are indeed different things. This has been reworded as follows:  Original: Those who answered ‘yes’ to both questions were considered to have an LLSI for the purposes of this study.  Revised: Those who answered ‘yes’ to both questions were counted as having the second outcome of current treatment for depression or serious anxiety. |
| More broadly, I would encourage revisiting the definitions of LLSI and the measured variables to ensure readers can clearly understand how they are operationalized. As it currently reads, there’s a risk of confounding the association between chronic illness and distress if mental health diagnosis or treatment is part of the LLSI classification, which would be concerning from a validity of outcomes perspective. If this isn’t the case, tightening the definitions and explicitly outlining the variables measured would address this concern. | We think this concern relates to the (now corrected) incorrect wording in the item above, which would have implied strong confounding between the exposure and outcome – given that the second outcome was incorrectly stated to be evidence for exposure. |
| Overall, I think this is a valuable piece of research, and with some adjustments to definitions, structure, and flow, it will be even stronger. | We thank the reviewer again for identifying these issues which made the exposure and outcome criteria unclear. |
| **Reviewer 2 comment** | **Response to reviewer 2** |
| In #4 above I selected "no" when asked about the writing standard. In fact, the authors do write well, but there are areas where they can explain their purpose more clearly. For example, they use a definition of LLSI (limiting and long-lasting illness) in different ways from the originally presented definition. | We believe this may refer to line 134 as noted by reviewer 1, where there was a mistake in wording, mixing up the exposure (LSLI) with the second outcome of treatment for depression or serious anxiety. These are indeed different things. This has been reworded as follows:  Original: Those who answered ‘yes’ to both questions were considered to have an LLSI for the purposes of this study.  Revised: Those who answered ‘yes’ to both questions were counted as having the second outcome of current treatment for depression or serious anxiety. |
| Additionally, as the data base is from a large UK study, the authors refer to UK codes that represent, as an example, various work-level advances (NVK). These are not explained, and readers outside of UK will not likely know what this refers to. So my primary concern as that the paper at times does not clearly explain some of it's intentions, and does not always explain terminologies. | This is a helpful comment. Regarding NVQ qualification levels specifically, we have now provided guidance on meaning:  “highest parental qualification, coded according to National Vocational Qualification (NVQ) or equivalent levels, i.e. (coded as 0=none or overseas qualification, 1=NVQ level 1 (a few low grades at school qualifications typically gained at age 16), 2=NVQ level 2 (higher grades at school qualification typically gained at age 16, 3=NVQ level 3 (school qualifications typically gained at age 18), 4=NVQ level 4 (further education or undergraduate degree level qualifications), 5=NVQ level 5 (higher than degree – e.g. masters level/doctoral - qualifications)”  As the sentence setting out covariates was particularly long, we have also now split this into a bulleted list for clarity.  We have reviewed the paper for other similar UK-centric terminology. |
| The comments ask for basic changes. Overall it is a good manuscript from my perspective.  Thank you for the opportunity to review this manuscript! | Many thanks for your helpful review. |
